# Calorie restriction reduces rDNA recombination independently of rDNA silencing

**DOI:** 10.1111/j.1474-9726.2009.00514.x

**Published:** 2009-12

**Authors:** Michèle Riesen, Alan Morgan

**Affiliations:** Department of Physiology, School of Biomedical Sciences, University of LiverpoolCrown St., PO Box 147, Liverpool L69 3BX, UK

**Keywords:** aging, dietary restriction, longevity, Sir2, telomere, yeast

## Abstract

Calorie restriction (CR) extends lifespan in yeast, worms, flies and mammals, suggesting that it acts via a conserved mechanism. In yeast, activation of the NAD-dependent histone deacetylase, Sir2, by CR is thought to increase silencing at the ribosomal DNA, thereby reducing the recombination-induced generation of extrachromosomal rDNA circles, hence increasing replicative lifespan. Although accumulation of extrachromosomal rDNA circles is specific to yeast aging, it is thought that Sirtuin activation represents a conserved longevity mechanism through which the beneficial effects of CR are mediated in various species. We show here that growing yeast on 0.05 or 0.5% glucose (severe and moderate CR, respectively) does not increase silencing at either sub-telomeric or rDNA loci compared with standard (2% glucose) media. Furthermore, rDNA silencing was unaffected in the *hxk2Δ*, *sch9Δ* and *tor1Δ* genetic mimics of CR, but inhibited by *FOB1* deletion. All these interventions extend lifespan in multiple yeast backgrounds, revealing a poor correlation between rDNA silencing and longevity. In contrast, CR and deletion of the *FOB1*, *HXK2*, *SCH9* and *TOR1* genes, all significantly reduced rDNA recombination. This silencing-independent mechanism for suppressing rDNA recombination may therefore contribute to CR-mediated lifespan extension.

## Introduction

In recent years, there has been an increasing recognition that similar fundamental cellular processes underlie aging in all eukaryotes (Guarente & Kenyon, 2000). Perhaps the most persuasive evidence that aging has a common cellular basis across phyla comes from studies of dietary limitation, often termed calorie restriction (CR) (Bishop & Guarente, 2007). Although data are not available for humans, CR has been known for over 70 years to increase lifespan in rodents, with more recent studies confirming the generality of this phenomenon in both vertebrates and invertebrates (Merry, 2005; Partridge *et al.*, 2005). Despite this, the precise molecular mechanism by which CR increases longevity in diverse species remains unclear.

Studies using the budding yeast, *Saccharomyces cerevisiae*, have been at the forefront of recent efforts to understand the molecular mechanism of action of CR. Yeast lifespan can be measured in two ways. Replicative lifespan is defined as the number of buds produced by an individual yeast mother cell and is independent of calendar time (Mortimer & Johnston, 1959; Muller *et al.*, 1980); in contrast to the alternative measure of the duration of viability in stationary phase, termed chronological lifespan (MacLean *et al.*, 2001). Reducing the concentration of glucose in yeast growth media from the standard 2% to 0.5% or below increases both replicative and chronological lifespan irrespective of genetic background and has been suggested to be a model of CR (Jiang *et al.*, 2000; Lin *et al.*, 2000; Fabrizio *et al.*, 2005; Smith *et al.*, 2007).

**Fig. 1:**
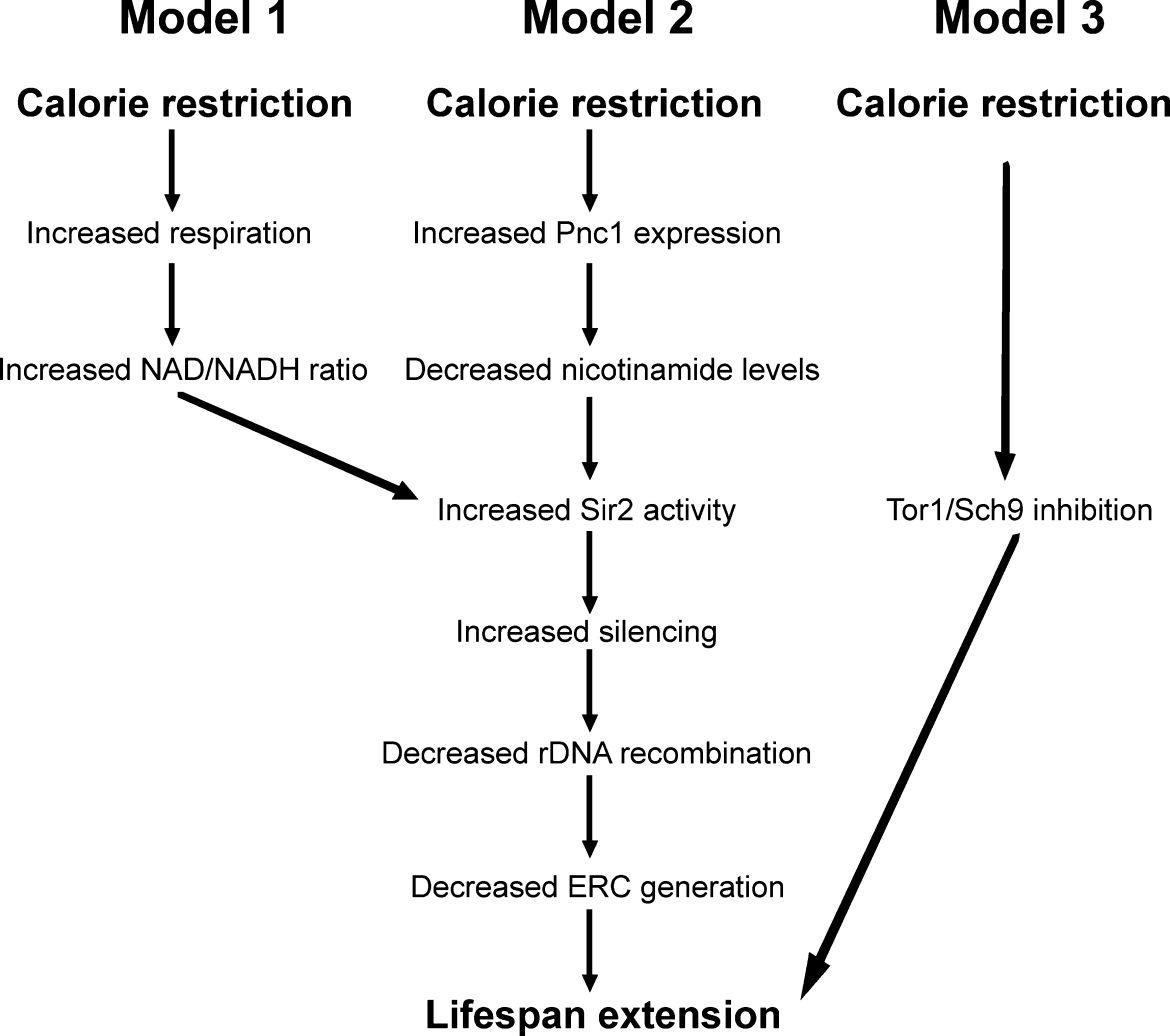
Proposed mechanisms of lifespan extension by calorie restriction. Models 1 and 2 suggest that CR up-regulates Sir2 activity, which leads to increased gene silencing and decreased rDNA recombination. Consequently, less ERCs are formed, which extends the yeast replicative lifespan. Both models differ only in the events upstream of Sir2 activation. Model 1 suggests that the increase in respiration in response to nutrient deprivation modulates the NAD^+^/NADH ratio in favour of NAD^+^, whereas model 2 explains the increase in Sir2-activity via a rise in Pnc1 levels and consequently the degradation of the endogenous Sir2 inhibitor, nicotinamide. Model 3 bypasses Sir2-dependent gene silencing altogether and puts forward the Tor1/Sch9 kinase pathways as links between calorie restriction and lifespan extension; although the downstream mechanism has not been fully determined, this is postulated to involve reduced ribosomal protein synthesis.

Although the effect of glucose limitation in extending yeast replicative lifespan is not disputed, its mechanism of action is the subject of considerable controversy and heated debate. Three main models have been suggested, two of which centre on the evolutionarily conserved NAD-dependent histone deacetylase, Sir2 (Fig. 1). Initially, it was proposed that CR causes a metabolic shift away from fermentation and towards respiration, resulting in an increased NAD:NADH ratio, and thus increased Sir2 activity (Lin *et al.*, 2000, 2002, 2004) (model 1). Alternatively (model 2), it was suggested that CR does not alter the NAD:NADH ratio, but rather acts by increasing expression of Pnc1, which degrades the endogenous Sir2 inhibitor nicotinamide, thus increasing Sir2 activity (Anderson *et al.*, 2003). In both models, activation of Sir2 leads to increased rDNA silencing and a consequent reduction in recombination between rDNA repeats; this reduces the formation of extrachromosomal rDNA circles (Sinclair & Guarente, 1997) and so retards aging.

In contrast, the observation that CR could extend lifespan in some *sir2* deletion strains (Jiang *et al.*, 2002; Kaeberlein *et al.*, 2004) led to the proposal of a third mechanism (Fig. 1, model 3). This postulates that CR extends lifespan in a Sir2-independent manner via inhibition of the Tor and Sch9 kinase signalling pathways (Kaeberlein *et al.*, 2004, 2005c). In this model, the downstream molecular mechanism effecting longevity is not entirely clear, but may be related to reduced ribosomal protein biogenesis (Kaeberlein *et al.*, 2005c; Steffen *et al.*, 2008). Controversially, it has recently been claimed that such Sir2-independent effects of CR are in fact due to activation of Sir2 homologues and so simply result in the same increase in rDNA silencing invoked in models 1 and 2 (Lamming *et al.*, 2005; Medvedik *et al.*, 2007) [but see (Kaeberlein *et al.*, 2006)].

Despite the popularity of models 1 and 2, there is surprisingly little published data demonstrating that CR does in fact increase transcriptional silencing at the rDNA locus (Lin *et al.*, 2002). Indeed, most published work has exploited ‘genetic mimics’ of CR and assayed rDNA recombination, rather than directly analysing silencing in response to reduced glucose levels. Furthermore, it has recently been shown that severe CR has no effect on transcriptional silencing at telomeres, which is a similarly Sir2-dependent process (Kaeberlein *et al.*, 2005a,c;). We therefore set out to determine the effect of CR and genetic mimics of CR on rDNA silencing and recombination. We found that rDNA silencing was unaffected by CR or by deletion of *HXK2*, *SCH9* or *TOR1*, whereas deletion of *FOB1* greatly inhibited rDNA silencing. In contrast, all of these well established lifespan-extending interventions reduced rDNA recombination. Taken together, these data are consistent with a silencing-independent mechanism for suppression of rDNA recombination in CR-mediated lifespan extension.

## Results

**Fig. 2:**
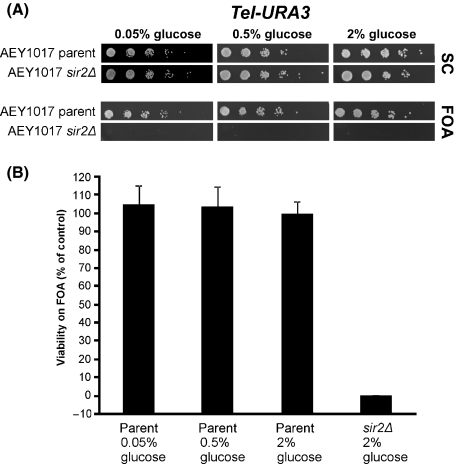
Calorie restriction does not affect subtelomeric gene silencing. (A) Qualitative silencing assay. The telomeric silencing reporter parent strain, AEY1017, and its isogenic *sir2Δ* strain were grown up in YPD, then diluted in H_2_O and serially spotted out on synthetic complete media (*SC*) and SC + FOA media (*FOA*). Spots correspond to OD_600_ = 1 (left) then serial 1 in 5 dilutions to the right. Plates were imaged after 2 (0.5% and 2% glucose) and 3 days (0.05% glucose) at 30 °C. Reduced growth on FOA compared with SC indicates reduced silencing. (B) Quantitative silencing assay. Serial dilutions of overnight cultures were spread on plates and colonies were counted after 2 (0.5% and 2% glucose) or 3 days (0.05% glucose). Silencing is proportional to viability on FOA media, which was calculated by dividing the numbers of colonies on FOA plates by the total numbers of colonies on −ura and FOA plates combined. 62 000 colonies were counted in total. The graph shows pooled data normalized to the 2% glucose control value, expressed as mean + standard error of the mean. No statistically significant difference between the conditions was found.

If CR acts via Sir2 activation (Fig. 1, models 1 and 2), then this would be predicted to result in increased silencing at subtelomeric loci. However, it has recently been claimed that severe CR imposed by growth on 0.05% glucose has no effect on telomeric silencing in the PSY316 yeast strain (Kaeberlein *et al.*, 2005c). We therefore investigated the effects of both moderate (0.5% glucose) and severe (0.05% glucose) CR on telomeric silencing using a well established reporter strain, AEY1017, which has a *URA3* marker integrated into a subtelomeric region of chromosome VII (Meijsing & Ehrenhofer-Murray, 2001). Expression of the *URA3* gene from nonsilenced genomic loci enables growth on media lacking uracil, but prevents growth on media containing FOA, because of conversion to the toxic product, 5-fluorouracil. However, integration of *URA3* into subtelomeric regions results in partial silencing of the reporter gene and hence the unusual ability to grow both on media lacking uracil and on media containing FOA. Deletion of the *SIR2* gene inhibits telomeric silencing, resulting in increased expression of the *URA3* reporter gene, which is routinely visualized as decreased growth on FOA. Indeed, no growth was observed on FOA plates for the *sir2* deletion strain (Fig. 2A), thus confirming the validity of this approach for assaying in vivo Sir2 activity. In contrast to the profound effect of *sir2* deletion on silencing, no obvious difference in growth on FOA plates was seen between 2%, 0.5% and 0.05% glucose (Fig. 2A).

Although CR had no obvious effect on telomeric silencing in spot tests, subtle effects could be missed in this simple visual assay. We therefore performed quantitative silencing assays by counting the number of individual colonies formed on selective FOA plates relative to the total number of colonies on nonselective media, as previously described (Kaeberlein *et al.*, 2005c). Increased silencing should result in increased viability in this assay, but over a series of 6 independent experiments totalling more than 60 000 colonies counted, we found no significant difference between 2%, 0.5% and 0.05% glucose (Fig. 2B). Thus, these quantitative silencing assays confirmed the findings from spot test assays: neither moderate nor severe CR affects telomeric silencing.

**Fig. 3:**
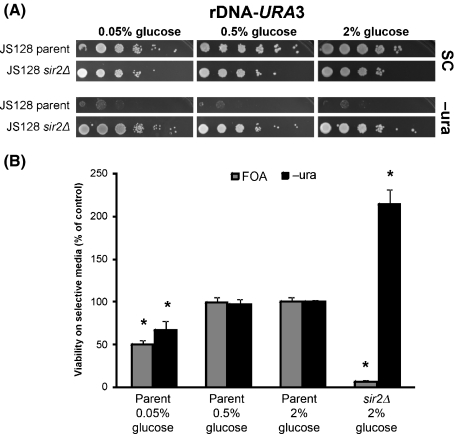
Calorie restriction does not affect gene silencing in the rDNA. (A) Qualitative silencing assay. The rDNA silencing reporter parent strain, JS128, and its isogenic *sir2Δ* strain were grown up in YPD, then diluted in H_2_O and serially spotted out on synthetic complete media (*SC*) and SC − ura media (−*ura*). Spots correspond to OD_600_ = 1 (left) then serial 1 in 5 dilutions to the right. Plates were imaged after 2 (0.5% and 2% glucose) and 3 days (0.05% glucose) at 30 °C. Reduced growth on −ura compared with SC indicates increased silencing in this assay. (B) Quantitative silencing assay. Serial dilutions of overnight cultures were spread on plates and colonies were counted after 2 (0.5% and 2% glucose) or 3 days (0.05% glucose). Silencing is inversely proportional to viability on −ura media, which was calculated by dividing the numbers of colonies on −ura plates by the numbers of colonies on SC plates (black bars). Viability on FOA was assessed in parallel by dividing the numbers of colonies on FOA plates by numbers of colonies on SC plates (grey bars). 63 000 colonies were counted in total. The graph shows pooled data normalized to the 2% glucose control value, expressed as mean + standard error of the mean. Statistical analysis was performed using Student’s *t*-tests (*P<0.05).

Although CR had no effect on telomeric silencing, it nevertheless remained possible that rDNA silencing was indeed increased. We therefore utilized the widely used rDNA reporter strain, JS128, which has a *URA3* marker integrated into the nontranscribed spacer region 1 of the rDNA (Smith & Boeke, 1997). Again, partial silencing of this reporter gene enables growth on media lacking uracil and media containing FOA. Deletion of the *SIR2* gene inhibits rDNA silencing, resulting in increased expression of the *URA3* reporter gene, which is routinely visualized in such rDNA reporter strains as increased growth on −ura plates (Smith & Boeke, 1997; Smith *et al.*, 2007) (Fig. 3A). If CR increases rDNA silencing, this should be seen as decreased growth on −ura plates relative to nonselective media. However, no gross effects were seen in spot tests using 0.5% and 0.05% glucose (Fig. 3A). To quantify these effects, a similar approach was taken to that used for the telomeric reporter assays described above, where the number of viable colonies on −ura plates relative to nonselective media was calculated. Over a series of three independent experiments with over 50 000 colonies counted, no significant difference in rDNA silencing was seen between 2% and 0.5% glucose (Fig. 3B). However, a partial reduction in viability on −ura plates was noted at 0.05% glucose. Such reduced growth on −ura plates could either be due to increased rDNA silencing or to a synthetic growth phenotype caused by the simultaneous imposition of the double stress of limiting glucose and uracil levels. To control for the latter, duplicate assays were performed in parallel using a different selective media, FOA, where increased silencing could not correlate with decreased colony growth (Fig. 3B, grey bars). This revealed a similar growth inhibition at 0.05% glucose (but not at 0.5% glucose), indicating that the partial reduction in viability on −ura media observed is not due to increased rDNA silencing. Confirmation of the artefactual nature of this reduced growth at 0.05% glucose was evident by comparison with a positive control *sir2Δ* strain (Fig. 3B), which exhibited the expected reciprocal relationship between growth on the two media (increased on −ura, decreased on FOA). Thus, both spot test and quantitative silencing assays indicate that moderate and severe CR have no effect on rDNA silencing.

**Fig. 4:**
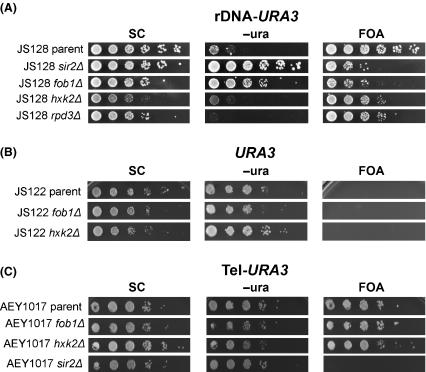
Effects of lifespan-extending mutations on rDNA silencing. (A) The rDNA silencing reporter parent strain, JS128, and isogenic deletion strains *hxk2Δ* and *fob1Δ*, were grown up in YPD. These were then diluted in H_2_O and serially spotted out on synthetic complete media (*SC*), SC − ura media (−*ura*) and SC + FOA media (*FOA*) containing 2% glucose. Spots correspond to OD_600_ = 1 (left) then serial 1 in 5 dilutions to the right. Plates were imaged after 2 days at 30 °C. Reduced growth on −ura compared with SC indicates increased silencing in this assay. (B) The JS122 control strain, where *URA3* is integrated in a nonsilenced locus, and isogenic deletion strains comprising *hxk2Δ* and *fob1Δ*, were grown up in YPD and plated exactly as above. (C) The telomeric silencing reporter parent strain, AEY1017, and its isogenic deletion strains, *hxk2Δ* and *fob1Δ*, were grown up in YPD, plated as above and imaged after 3 days. Reduced growth on FOA is a readout of reduced silencing in this assay.

The correlation between increased rDNA silencing and longevity is integral to Sirtuin-dependent models of yeast lifespan extension. As we found no effect of CR on rDNA silencing, we investigated whether deletion mutations that reproducibly extend lifespan in multiple yeast background strains alter rDNA silencing. Deletion of *HXK2*, one of three hexokinases in yeast, limits the entry of glucose into glycolysis and hence is widely used as a genetic mimic of CR acting upstream of Sir2/Tor/Sch9. Indeed, like CR, *HXK2* deletion has been shown to increase lifespan in multiple yeast genetic backgrounds (Lin *et al.*, 2000; Kaeberlein *et al.*, 2005b). Deletion of *FOB1* is thought to increase lifespan by reducing rDNA recombination [and hence extrachromosomal rDNA circle (ERC) generation] downstream of Sir2 (Defossez *et al.*, 1999; Kaeberlein *et al.*, 2005b). In Fig. 4A, it can be seen that the *hxk2Δ* strain behaves similarly to the JS128 parent strain on all media, indicating no major effect on rDNA silencing. However, the *fob1Δ* strain exhibited enhanced growth on −ura plates, and decreased growth on FOA plates, similar to the *sir2Δ* strain, indicating a loss of rDNA silencing. In contrast, deletion of *RPD3*, which is known to increase rDNA silencing in a Sir2-dependent manner (Sun & Hampsey, 1999), gave the expected decrease in growth on −ura plates, thus confirming that this assay is able to detect such increases in rDNA silencing. To check if these effects were rDNA-specific, we deleted *FOB1* and *HXK2* in a control strain (JS122) where the *URA3* reporter gene is integrated in a nonsilenced genomic locus. Here, there was no difference in growth on selective media between the parent strain and its isogenic mutants: all grew similarly on −ura plates, none grew on FOA (Fig. 4B). Finally, we investigated whether telomeric silencing was similarly affected by creating deletion mutants in the AEY1017 strain. In spot test assays, the *hxk2Δ* strain was similar to its isogenic parent strain and to a *fob1Δ* strain (Fig. 4C). Therefore, the *hxk2Δ* genetic mimic of CR, like CR itself, has little effect on silencing at rDNA or telomeric loci, whereas deletion of *FOB1* specifically inhibits rDNA silencing.

**Fig. 5:**
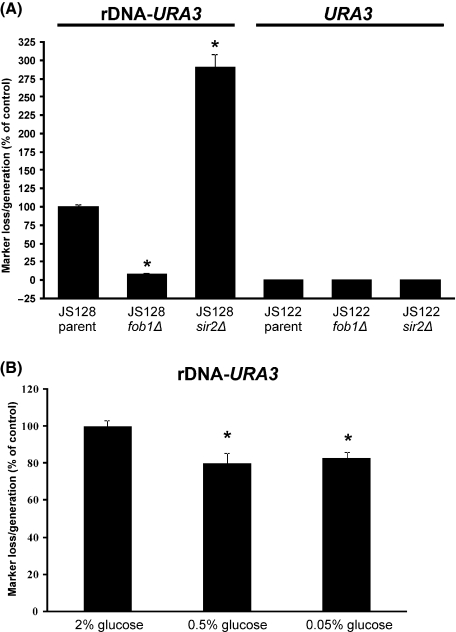
Calorie restriction reduces rDNA recombination. The rDNA silencing reporter parent strain, JS128, the control JS122 strain, and isogenic *fob1Δ* and *sir2Δ* strains were serially cultured in YP media containing the indicated glucose concentrations for approximately 120 generations. These were then spread onto YPD plates and then replica plated onto both SC and −ura plates. Marker loss per generation was calculated by dividing the number of colonies that did not grow on −ura plates by the total number of colonies on replica SC plates and dividing this value by the total number of generations. In total, 73 000 colonies were counted for (A) and 69 000 colonies for (B). Each graph represents pooled data normalized to the appropriate JS128 parent 2% glucose control value, expressed as mean + standard error of the mean. Statistical analysis was performed using Student’s *t*-tests (*P<0.05).

Despite these different effects on silencing, both CR and deletion of *FOB1* have been reported to extend lifespan via inhibition of rDNA recombination. We therefore assayed mitotic rDNA recombination by monitoring the frequency of *URA3* marker loss after serial culturing for approximately 120 generations (Dror & Winston, 2004). Over a series of experiments, the mean frequency of marker loss in the parent JS128 strain at 2% glucose was 1.3 × 10^−3^ per generation, similar to previously reported values for other strains (Merker & Klein, 2002; Lamming *et al.*, 2005). Deletion of *FOB1* reduced the frequency of *URA3* marker loss by around 90%, whereas deletion of *SIR2* caused an approximately threefold increase in marker loss (Fig. 5A), consistent with the known pro- and anti-recombinase functions of Fob1 and Sir2, respectively, at the rDNA (Merker & Klein, 2002). These effects on *URA3* marker loss were specific for the rDNA locus, as no marker loss was observed over 120 generations in the JS122 parent strain where *URA3* is integrated outside the rDNA, or in its isogenic JS122 *fob1Δ* and *sir2Δ* strains (Fig. 5A). These data confirmed the validity of assaying the frequency of *URA3* marker loss in the JS128 strain as a readout of rDNA recombination rate, thus enabling the effects of CR to be determined. Both moderate (0.5% glucose) and severe (0.05% glucose) CR produced a significant reduction in rDNA recombination of around 20% (Fig. 5B). Therefore, both CR and *FOB1* deletion act via a silencing-independent mechanism to reduce rDNA recombination.

**Fig. 6:**
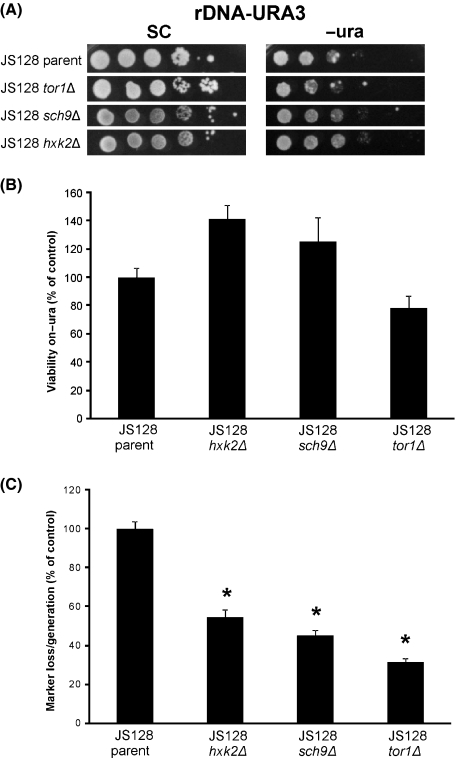
Genetic mimics of calorie restriction reduce rDNA recombination independently of rDNA silencing. (A) Qualitative silencing assay. The rDNA silencing reporter parent strain, JS128, and its isogenic *sch9Δ*, *tor1Δ* and *sir2Δ* strains were grown up in YPD, then diluted in H_2_O and serially spotted out on synthetic complete media (*SC*) and SC − ura media (−*ura*). Spots correspond to OD_600_ = 1 (left) then serial 1 in 5 dilutions to the right. Plates were imaged after 2 (0.5% and 2% glucose) and 3 days (0.05% glucose) at 30 °C. Reduced growth on −ura compared with SC indicates increased silencing in this assay. (B) Quantitative silencing assay. Threefold serial dilutions of overnight cultures of JS128 and its isogenic *hxk2Δ*, *sch9Δ* and *tor1Δ* strains were spread on plates and colonies were counted after 2 days. Silencing is expressed as a function of viable clones on −ura by dividing the numbers of colonies on −ura by numbers of colonies on SC. 29 000 colonies were counted in total. The graph shows pooled data normalized to the parent 2% glucose control value, expressed as mean + standard error of the mean. Statistical analysis was performed using Student’s *t*-tests (*P<0.05). (C) Recombination assay. JS128 and its isogenic *hxk2Δ*, *sch9Δ* and *tor1Δ* strains were serially cultured in YPD for approximately 120 generations. These were then spread onto YPD plates and then replica plated onto both SC and −ura plates. Marker loss per generation was calculated by dividing the number of colonies that did not grow on −ura plates by the total number of colonies on replica SC plates and dividing this value by the total number of generations. 43 000 colonies were counted in total. The graph shows pooled data normalized to the parent 2% glucose control value, expressed as mean + standard error of the mean. Statistical analysis was performed using Student’s *t*-tests (*P<0.05).

Finally, we investigated whether the Sir2-independent Sch9/Tor1-mediated longevity pathway of model 3 might also impact on rDNA silencing and recombination. Deletion of *SCH9* appeared to slightly improve growth on −ura media relative to nonselective media in qualitative spot test assays (Fig. 6A), similar to the general CR mimic *hxk2Δ* (Fig. 4A, 6A); whereas, deletion of *TOR1* appeared to slightly decrease growth on −ura plates (Fig. 6A). Although these small effects were also apparent in quantitative silencing assays (Fig. 6B), the differences in viability on −ura plates between the various deletion mutants and the isogenic parent strain were not statistically significant. Therefore, like CR itself, genetic mimics of CR do not cause significant increases in rDNA silencing. In contrast, the *hxk2Δ*, *sch9Δ* and *tor1Δ* strains all exhibited significantly reduced rDNA recombination (Fig. 6C), reinforcing the notion that various lifespan-extending interventions can reduce rDNA recombination independently of rDNA silencing.

## Discussion

Reducing the concentration of glucose from the standard 2% to 0.5% or below has been reported to extend replicative lifespan in all yeast strains examined (Jiang *et al.*, 2000; Lin *et al.*, 2000; Kaeberlein *et al.*, 2004; Lamming *et al.*, 2005). Likewise, deletion of *SIR2* shortens replicative lifespan and overexpression of Sir2 extends replicative lifespan in most strains (Kaeberlein *et al.*, 1999, 2004; Kim *et al.*, 1999; Anderson *et al.*, 2003). While there is little doubt that both CR and Sir2 are powerful and near-universal modulators of longevity, debate has raged over whether the two are part of a single mechanism or represent separate pathways (Kaeberlein & Powers, 2007). Sir2 is an attractive candidate for a central mediator of CR, as its NAD-dependent histone deacetylase activity could potentially sense CR-induced increases in the NAD:NADH ratio or decreases in nicotinamide levels and transduce these into increased rDNA silencing and consequently reduced rDNA recombination. The reported lack of effect of severe CR on telomeric silencing (Kaeberlein *et al.*, 2005c), which we have confirmed here and extended to moderate CR, argues against this idea, as does the observation that CR can extend lifespan in some *SIR2* deletion strains (Jiang *et al.*, 2002; Kaeberlein *et al.*, 2004). Nevertheless, this does not rule out the possibility that CR acts specifically on the rDNA to increase silencing. Indeed, a recent screen for genes that increase rDNA silencing in the absence of Sir2 has claimed that Sir2-independent lifespan extension by CR in fact proceeds via activation of the Sir2 homologue, Hst2, and so ultimately works by the same mechanism of increased rDNA silencing/decreased rDNA recombination (Lamming *et al.*, 2005). However, the data presented here strongly suggest that CR has no effect on rDNA silencing, and thus acts via a pathway(s) that does not involve activation of Sir2 or its homologues.

How can we reconcile our data with earlier work that established models 1 and 2? While there is no doubt that Sir2 histone deacetylase activity is of primary importance in establishing rDNA silencing, a survey of the literature reveals surprisingly little data directly demonstrating that CR affects rDNA silencing. Indeed, to our knowledge, the only such data is Fig. 1d in (Lin *et al.*, 2002), where the result of a qualitative assay using *MET15* as a reporter gene inserted in the rDNA was presented. The increased brown colouration of this strain on 0.5% glucose compared with 2% glucose was consistent with an effect of moderate CR increasing rDNA silencing. However, such assays are not quantifiable and are subject to interpretations over ‘shades of brownness’. Furthermore, similar effects of CR on *MET15* reporter gene expression are observed when the marker is integrated into nonsilenced regions of the genome, suggesting that the increased pigmentation observed is a metabolic effect unrelated to silencing (Smith *et al.*, 2009). The data presented here, using both qualitative and quantitative assays of *URA3* reporter gene expression, strongly argue that CR has no effect on rDNA silencing. As CR has no effect on telomeric silencing either (this study and Kaeberlein *et al.*, 2005c), it seems unlikely that activation of Sir2 (or its homologues) occurs in response to CR. Furthermore, rDNA silencing is not significantly affected in the CR genetic mimics, *hxk2Δ*, *sch9Δ* and *tor1Δ*; whereas *fob1Δ* strains actually exhibit greatly reduced rDNA silencing. As CR and all these deletion mutants have been shown to increase lifespan in various genetic backgrounds, these observations further emphasize the lack of correlation between rDNA silencing and lifespan, and so make it unlikely that Sirtuin-mediated rDNA silencing is crucial for longevity.

What implications do our data have for the competing theories of CR outlined in Fig. 1? It is important to note that although our data clearly argue against the essential role for Sir2 activation and increased rDNA silencing implicit in models 1 and 2, they are nevertheless consistent with the proposal that CR extends lifespan by reducing rDNA recombination, albeit via a silencing-independent mechanism. Indeed, the observation that *FOB1* deletion inhibits rDNA silencing (this study and Huang & Moazed, 2003), clearly demonstrates that these two processes can be uncoupled; as *FOB1* deletion strongly decreases rDNA recombination (this study and Defossez *et al.*, 1999; Kobayashi *et al.*, 1998). As Fob1 is a component of the RENT complex that targets Sir2 to the nucleolus (Huang & Moazed, 2003), the consequent loss of Sir2 from the rDNA explains the reduced silencing in *FOB1* deletion strains, but this in turn inevitably suggests that the decrease in rDNA recombination is Sir2- and silencing-independent. It has previously been shown that rDNA recombination is reduced by moderate CR, rapamycin and by the putative CR genetic mimic *cdc25-10* (Lin *et al.*, 2000; Lamming *et al.*, 2005; Medvedik *et al.*, 2007). We report here that both moderate and severe CR, as well as deletion of the *HXK2*, *SCH9* and *TOR1* genes all reduce rDNA recombination. Thus, it may be that models 1 and 2 are essentially correct in that CR ultimately extends lifespan, at least in part, by decreasing rDNA recombination, but that this occurs by an (unknown) Sirtuin-independent process. In both models, the longevity associated with reduced rDNA recombination is attributed to reduced ERC generation. While this remains a plausible mechanism, it is interesting to note that in *fob1/sir2* double mutants, which have extremely low ERC levels, CR increases lifespan and reduces rDNA recombination (Kaeberlein *et al.*, 1999, 2004; Lamming *et al.*, 2005). In addition, lifespan reduction is associated with increased rDNA recombination in the face of unchanged ERC levels in *sgs1*, *hpr1* and *dna2* mutants (Heo *et al.*, 1999; McVey *et al.*, 2001; Hoopes *et al.*, 2002; Mankouri *et al.*, 2002; Merker & Klein, 2002). Further work is required to determine if the CR-induced reduction in rDNA recombination impacts on longevity via ERCs or a different mechanism.

Although the Sir2-independent, Sch9/Tor1-regulated model 3 centres upon reduced ribosomal protein synthesis as the downstream mechanism of lifespan extension (Kaeberlein *et al.*, 2005c; Steffen *et al.*, 2008), our data clearly show that these interventions also reduce rDNA recombination. Therefore, it is possible that CR and all its various genetic mimics extend lifespan, at least in part, by a common action in reducing rDNA recombination. The molecular mechanisms involved in this putative silencing-independent regulation of rDNA recombination are unknown. However, it is interesting to note that Tor1 physically interacts with the 35S rDNA promoter (Tsang *et al.*, 2003) and that its association with the rDNA is regulated by nutrient levels (Li *et al.*, 2006). In addition, it has recently been shown that localization of rDNA repeats to the inner nuclear membrane suppresses rDNA recombination independently of rDNA silencing (Mekhail *et al.*, 2008). Clearly, dissection of the molecular mechanisms involved in yeast lifespan extension by CR will require much further work in order to construct a single unifying model.

## Experimental procedures

### Chemicals and reagents

Materials for yeast culture were obtained from Sigma-Aldrich (Poole, UK) and Foremedium (Norwich, UK). 5-Fluoro-orotic acid (FOA) was obtained from Apollo Scientific (Stockport, UK). PCR primers were supplied by Sigma Genosys (Havenhill, UK), genomic DNA isolation kits were from Invitrogen (Paisley, UK); and PCR enzymes/reagents were from Promega (Southampton, UK). All other materials were obtained from Sigma-Aldrich.

### Strain construction

Telomeric silencing in this study was assessed using the telomeric *URA3* marker strain, AEY1017 (W303-1B; Matα*ade2-1 ura3-1 his3-11*,*15 leu2-3*, *112 trp1-1 can 1-100* TELVIIL::*URA3*) (Meijsing & Ehrenhofer-Murray, 2001). Silencing at rDNA was assessed using the rDNA *URA3* marker strain, JS128(S6) (JB740; MATα*his3Δ200 leu2Δ1 ura3-167* RDN1::Ty1::m*URA3*) (Smith & Boeke, 1997). As a control for silencing-independent effects, a strain with URA3 integrated at a nonsilenced locus was used, JS122 (JB740; MATα*his3Δ200 leu2Δ1 ura3-167* ??::Ty1::m*URA3*) (Smith & Boeke, 1997). Deletion strains with the appropriate open reading frame replaced by the KanMX4 cassette in the BY4741 *MAT*a haploid background were obtained from Invitrogen. Deletion cassettes for *FOB1*, *HXK*2, *SIR*2 and *TOR1* were PCR amplified from genomic DNA prepared from the respective BY4741 deletion strain. The SCH9 deletion cassette was PCR amplified from the pFA6KanMX4 plasmid (Longtine *et al.*, 1998) using appropriate primers. The resulting PCR products were used to transform each *URA3* reporter strain via the PCR-based disruption strategy (Schiestl & Gietz, 1989; Wach, 1996). Successful G418-resistant transformants were confirmed by PCR using gene-specific and KanMX primers (data not shown). Strains used in this study are listed in Table 1.

**Table 1:** Yeast strains used in this study

Strain	Genotype	Background (reference)
AEY1017 parent	*MAT*α*ade2-1 ura3-1 his3-11*,*15 leu2-3*, *112 trp1-1 can 1-100* TELVIIL*::URA3*	W303-1B (Meijsing & Ehrenhofer-Murray, 2001)
AEY1017 *sir2Δ*	AEY1017 *sir2Δ::kanMX4*	This study
AEY1017 *fob1Δ*	AEY1017 *fob1Δ::kanMX4*	This study
AEY1017 *hxk2Δ*	AEY1017 *hxk2Δ::kanMX4*	This study
JS128 parent	*MAT*α*his3Δ200 leu2Δ1 ura3-167 RDN1(NTS1)::Ty1-mURA3*	JB740 (Smith & Boeke, 1997)
JS128 *sir2Δ*	JS128 *sir2Δ::kanMX4*	This study
JS128 *fob1Δ*	JS128 *fob1Δ::kanMX4*	This study
JS128 *hxk2Δ*	JS128 *hxk2Δ::kanMX4*	This study
JS128 *sch9Δ*	JS128 *sch9Δ::kanMX4*	This study
JS128 *tor1Δ*	JS128 *tor1Δ::kanMX4*	This study
JS128 *rpd3Δ*	JS128 *rpd3Δ::kanMX4*	This study
JS122 parent	*MAT*α*his3Δ200 leu2Δ1 ura3-167 ????::Ty1-mURA3*	JB740 (Smith & Boeke, 1997)
JS122 *fob1Δ*	JS122 *fob1Δ::kanMX4*	This study
JS122 *hxk2Δ*	JS122 *hxk2Δ::kanMX4*	This study
JS122 *sir2Δ*	JS122 *sir2Δ::kanMX4*	This study

### Qualitative silencing assays (spottests)

For silencing assays, synthetic complete media (SC), uracil omission media (SC − ura) and FOA-supplemented media (SC + FOA) were prepared from 2× stock solutions and D-glucose added to 0.05%, 0.5% and 2% from a 50% stock solution. 5-Fluoro-orotic acid was prepared in DMSO and added to media to give a final concentration of 1 mg ml^−1^ and 1% DMSO final concentration. Single colonies were picked from a plate and grown overnight in 5 mL liquid YP media containing 2% glucose. The next morning, cultures were diluted to OD_600_ = 1 in sterile H_2_O in 1 mL. The adjusted cultures were serially diluted five times in sterile H_2_O at a ratio of 1:5 starting from OD_600_ = 1 in column 1 in a 96 well-plate to 100 μL final volume and plated with a replica plater onto SC, SC − ura and SC + FOA plates containing 0.05%, 0.5% and 2%, glucose and incubated at 30°C for 2 (0.5% and 2% glucose) to 4 days (0.05% glucose). Plates were imaged in a BioRad Universal Hood II Imager (BioRad, Hemel Hempstead, UK).

### Quantitative silencing assays

Cultures were grown overnight in 5 mL YPD, diluted with water to OD_600_ = 1 in 1 mL final volume. For silencing assays using the parent strains, cultures were then serially diluted in sterile H_2_O to 10^−3^, 5 × 10^−3^, 10^−4^, 5 × 10^−4^, and 100 μL of each dilution was plated on SC, SC − ura, or SC + FOA plates supplemented with the various glucose concentrations. The plates were incubated at 30°C for 2–4 days. For silencing assays using deletion strains, overnight liquid cultures were adjusted to OD_600_ = 1 in 1 mL, and serially diluted. On SC and SC − ura plates, 100 μL of a 10^−4^ dilution were plated, and on SC + FOA, 100 μL of a 10^−3^ dilution were plated. Colonies were counted either manually or with a BioRad Universal Hood II Imager and QuantityOne software (Biorad). Viability of the AEY1017 telomeric reporter strain on FOA medium is directly proportional to silencing activity (Meijsing & Ehrenhofer-Murray, 2001). Viability was calculated by dividing the number of colonies on FOA plates either by the number of colonies on SC plates (FOA/SC) or by the total number of colonies on −ura and FOA plates combined (FOA/−ura + FOA), both of which yielded similar results. Data from assays of six independently grown yeast clones were pooled and statistical analysis performed using Student’s *t*-tests. Viability of the JS128 rDNA reporter strain on −ura medium is inversely proportional to silencing activity (Smith & Boeke, 1997) and was calculated by dividing the number of colonies on −ura plates by the number of colonies on SC plates (-ura/SC). To control for synthetic growth effects of selective media at low glucose concentrations, duplicate assays were performed in parallel on FOA plates and viability calculated by dividing the number of colonies on FOA plates by the number of colonies on SC plates. Four platings per strain/condition were analysed for each rDNA silencing assay, and every assay was performed on at least two independently grown yeast clones. The resulting data were then pooled and statistical analysis performed using Student’s *t*-tests.

### rDNA recombination assays

Mitotic stability of the *URA3* gene in JS122 and JS128 strains was assayed using the method of Dror and Winston, 2004. Briefly, single colonies from -ura plates were grown to saturation in 10 mL YPD at 30 °C. At this point, the cultures were diluted 1:10 000 in 10 mL fresh YPD and grown to saturation once more. After nine such saturation passages (equivalent to approximately 120 generations), each culture was spread onto ten YPD plates at an appropriate dilution to yield 50–300 colonies/plate. After growth at 30 °C for 2–3 days, each plate was then replica plated onto both SC and −ura plates and grown for several days at 30 °C. Frequency of *URA3* marker loss by recombination was calculated by dividing the number of colonies that did not grow on −ura plates by the total number of colonies on replica SC or YPD plates. Marker loss per generation was calculated by dividing this value by the total number of mitotic divisions during the nine serial passages (Cole *et al.*, 2007). Ten platings per strain/condition were used for each recombination assay, and every assay was performed on at least two independently grown yeast clones. The resulting data were then pooled and statistical analysis performed using Student’s *t*-tests.
